# Tubule-specific deletion of *LincRNA-p21*ameliorates lipotoxic kidney injury

**DOI:** 10.1016/j.omtn.2021.10.029

**Published:** 2021-11-04

**Authors:** Bin Li, Joseph C.K. Leung, Loretta Y.Y. Chan, Hong-Yu Li, Wai-Han Yiu, Sarah W.Y. Lok, Rui Xue, Yi-Xin Zou, Wei Chen, Kar-Neng Lai, Sydney C.W. Tang

**Affiliations:** 1 Division of Nephrology, Department of Medicine, The University of Hong Kong, Hong Kong, People's Republic of China; 2 Department of Nephrology, The First Affiliated Hospital, Sun Yat-sen University, Guangzhou, People's Republic of China

**Keywords:** kidney, obesity, lipotoxicity, long non-coding RNA, high-fat diet, palmitic acid, inflammation, tubule cell, p53

## Abstract

Lipotoxicity has been implicated in the pathogenesis of obesity-related kidney damage and propagates chronic kidney injury like diabetic kidney disease; however, the underlying mechanisms have not yet been fully elucidated. To date, reduction of lipid acquisition and enhancement of lipid metabolism are the major, albeit non-specific, approaches to improve lipotoxic kidney damage. In the kidneys of high-fat diet (HFD)-fed mice and tubule cells cultured with palmitic acid (PA), we observed a dramatic upregulation of the long intergenic non-coding RNA-p21 (*LincRNA-p21*) through a p53-dependent mechanism. Kidney tubule cell-specific deletion of *LincRNA-p21* attenuated oxidative stress, inflammation, apoptosis, and endoplasmic reticulum stress, leading to reduction of histological and functional kidney injury despite persistent obesity and hyperlipidemia. Mechanistically, HFD- or PA-initiated lipotoxicity suppressed the phosphatidylinositol 3-kinase (PI3K)/protein kinase B (AKT)/mechanistic target of rapamycin (mTOR)/murine double minute 2 homolog (MDM2) signaling cascade to activate p53 and enhance the transcriptional activity of *LincRNA-p21*. Collectively, our findings suggest that the p53/*LincRNA-p21* axis is the downstream effector in lipotoxic kidney injury and that targeting this axis particularly in the kidney tubule could be a novel therapeutic strategy.

## Graphical abstract

**Figure:**
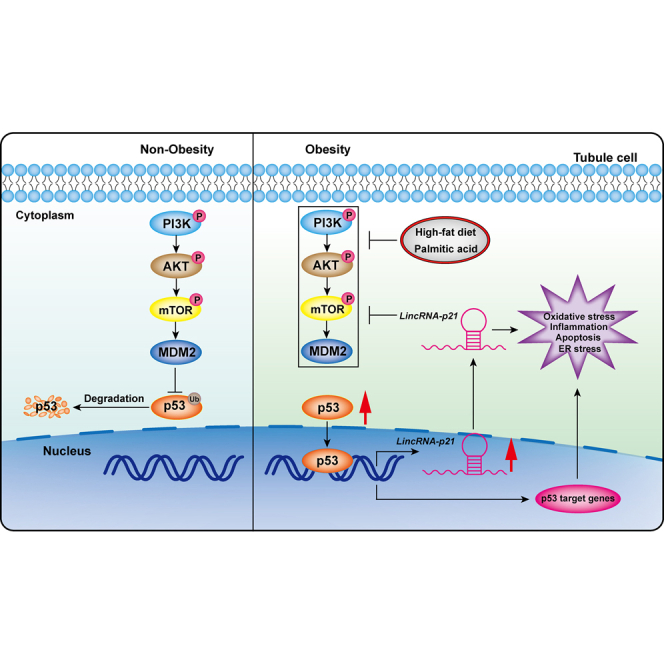

## Teaser

Our study observed a dramatic upregulation of *LincRNA-p21* through a p53-dependent mechanism in kidney tubule cells exposed to palmitic acid. Tubule cell-specific deletion of *LincRNA-p21* prevented histological and functional kidney injury induced by high-fat diet. These findings suggest p53/*LincRNA-p21* signaling axis as a potential therapeutic target for lipotoxic kidney injury.

## Introduction

Obesity has been considered as a major risk factor for the development of chronic kidney disease, independent of its association with hypertension, diabetes, and dyslipidemia.^1^ Epidemiological studies have shown that intake of excess saturated fatty acid is a principal lifestyle-related cause of hyperlipidemia and related diseases, including non-alcoholic fatty liver disease and obesity-related kidney injury. During high-fat diet (HFD) feeding, the influx of free fatty acids results in the accumulation of triglycerides in other non-adipose tissues, termed lipotoxicity, and this induces oxidative stress, inflammation, apoptosis, endoplasmic reticulum (ER) stress, and insulin resistance, thus leading to pathological and functional aberrations in multiple organs, including the kidney.^2^

In addition to the well-known role of tumor protein 53 (p53) in tumor suppression, a plethora of evidence suggests the extensive involvement of p53 in the regulation of energy metabolism and numerous metabolic processes like aging, obesity, and diabetes^3^ by manipulation of cell metabolism, energy homeostasis, and cell fate. In HFD-treated animals, p53 was functionally elevated in various tissues accompanied with aggravated chronic inflammation, senescence, and systemic insulin resistance.^3^^,^^4^ Pharmacologic inhibition or genetic ablation of p53 in mice fed with HFD impeded excess fat accumulation, weight gain, hepatosteatosis, and insulin resistance.^3^^,^^5^ How p53 activates lipid metabolism in response to HFD treatment is not fully understood and it will be imperative to dissect the tissue- and cell-specific actions of p53 and its downstream signaling pathways in order to unravel a new paradigm of therapy for the chronic inflammatory state in obesity.

Long intergenic non-coding RNAs (lncRNAs), a class of non-coding RNAs over 200 nucleotides in length, have been a center of intense research in recent years and recognized as important regulators involved in diverse obesity-associated biological and cellular processes.^6^ lncRNA p21 (*LincRNA-p21*) is one of the most-studied lncRNAs due to its ability to fine-tune p53-dependent transcriptional responses.^7^ The name of *LincRNA-p21* comes from its genetic location lying approximately 15 kb upstream from the Cyclin Dependent Kinase Inhibitor 1A (*Cdkn1a*, also called *p21*) gene on chromosome 17. Previous studies documented an integrative role of *LincRNA-p21* in a variety of cellular functions, including modulation of cellular proliferation, inflammation, apoptosis, cell-cycle arrest, ER stress, gene expression, and protein stability.^8^^,^^9^ Especially noteworthy is that silencing *LincRNA-p21* alleviated pathological changes in diabetic kidney.^10^ Nevertheless, little is known of whether *LincRNA-p21* participates in obesity-related kidney injury.

The current study aims to dissect the role and mechanism of the p53/*LincRNA-p21* signaling axis in the pathogenesis of lipotoxic injury arising from the chronic metabolic stress of excessive dietary fat consumption.

## Results

### Generating a tubule-specific *LincRNA-p21* knockout diet-induced obesity mouse model

**Figure 1:**
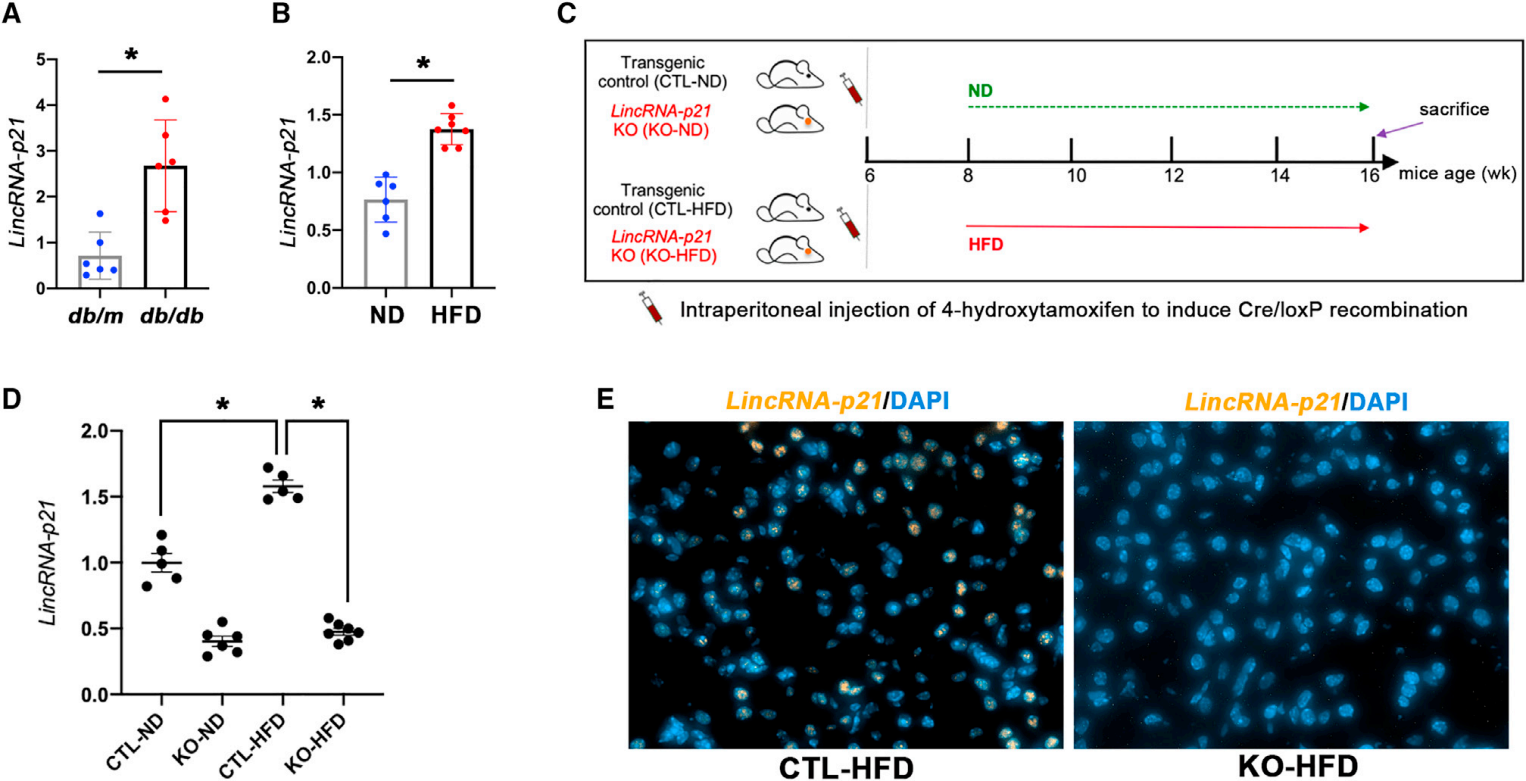
Characterization of *LincRNA-p21* in kidney (A) Increased expression of *LincRNA-p21* in renal cortex from *db/db* mice versus their nondiabetic *db/m* mice. (B) Increased expression of *LincRNA-p21* in renal cortex from diet-induced obese mice versus lean mice. (C) DIO protocol in tubule-specific *LincRNA-p21* KO mice and their CTL littermates. (D) Renal cortical expression of *LincRNA-p21* in KO or CTL mice fed with ND (KO-ND or CTL-ND) or HFD (KO-HFD or CTL-HFD) by qRT-PCR. (E) FISH showing the expression of nuclear *LincRNA-p21* (orange) in kidney section from CTL-HFD mice and absence of nuclear *LincRNA-p21* staining signal in KO-HFD mice. Nuclei were counterstained with 4′,6-diamidino-2-phenylindole (DAPI) (×400). Data represent the mean ± SEM for five to seven mice per group. ∗p < 0.05.

Elevated expression of *LincRNA-p21* was found in kidney tissue from obese *db/db* mice and HFD-fed obese mice (Figures 1A and 1B). To investigate the specific role of tubular *LincRNA-p21* in diet-induced obesity (DIO) and associated metabolic disorders, tubule-specific *LincRNA-p21* knockout (KO) mice and their control (CTL) littermates were generated (Figure S1) and fed normal diet (ND) or HFD for 8 weeks before sacrifice (Figure 1C). Gene KO was confirmed by quantitative real-time PCR (qRT-PCR) (Figure 1D). Tubule-specific *LincRNA-p21* KO mice and their CTL littermates had normal appearance and no difference in the size or morphology of kidney. HFD-induced upregulation of *LincRNA-p21* was reduced in KO mice as detected by qRT-PCR and fluorescence *in situ* hybridization (FISH) (Figures 1D and 1E).

### Tubule-specific *LincRNA-p21* deletion did not affect the development of obesity

**Figure 2:**
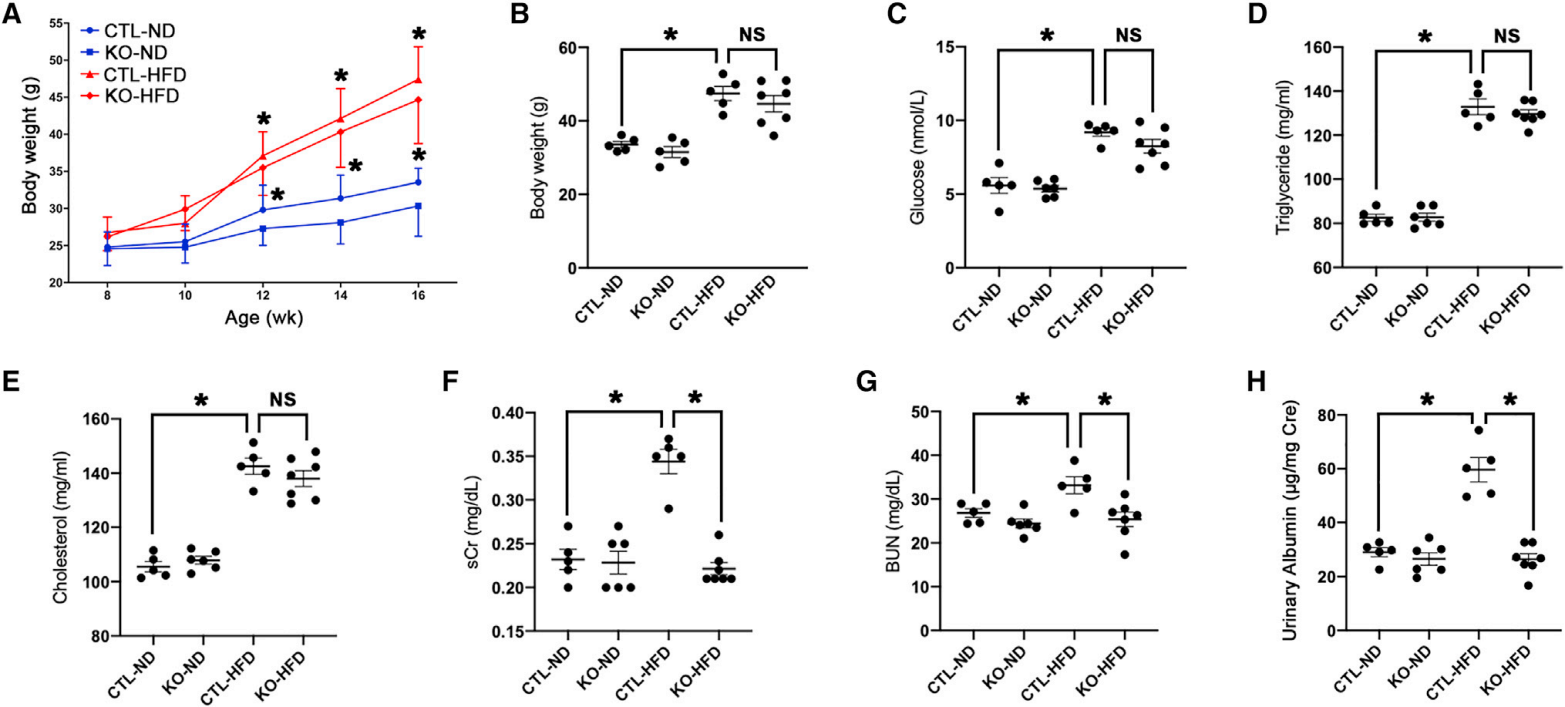
Effect of tubule-specific *LincRNA-p21* KO on biometabolic parameters in mice (A) Changes in BW over the experimental period. *LincRNA-p21* KO did not alter BW (B), blood glucose (C), serum triglyceride (D), and cholesterol (E) after 8 weeks of HFD. (F–H) *LincRNA-p21* KO significantly reduced sCr (F), BUN (G), and urinary albumin level (H) after 8 weeks of HFD. Data are mean ± SEM from each group (n ≥ 5); ∗p < 0.05; NS, statistically not significant.

HFD markedly increased BW in both KO and CTL mice from week 12 onward (Figure 2A), and no significant differences in body weight (BW) were observed for KO or CTL mice fed either ND or HFD (Figure 2B). Fasting blood glucose and serum lipids (Figures 2C–2E) were elevated by similar extents in both KO mice and CTL mice given HFD, indicating that tubular *LincRNA-p21* does not affect the metabolic profile in HFD-induced obesity.

### Tubule-specific *LincRNA-p21* deletion protected kidney from HFD-Induced injury

**Figure 3:**
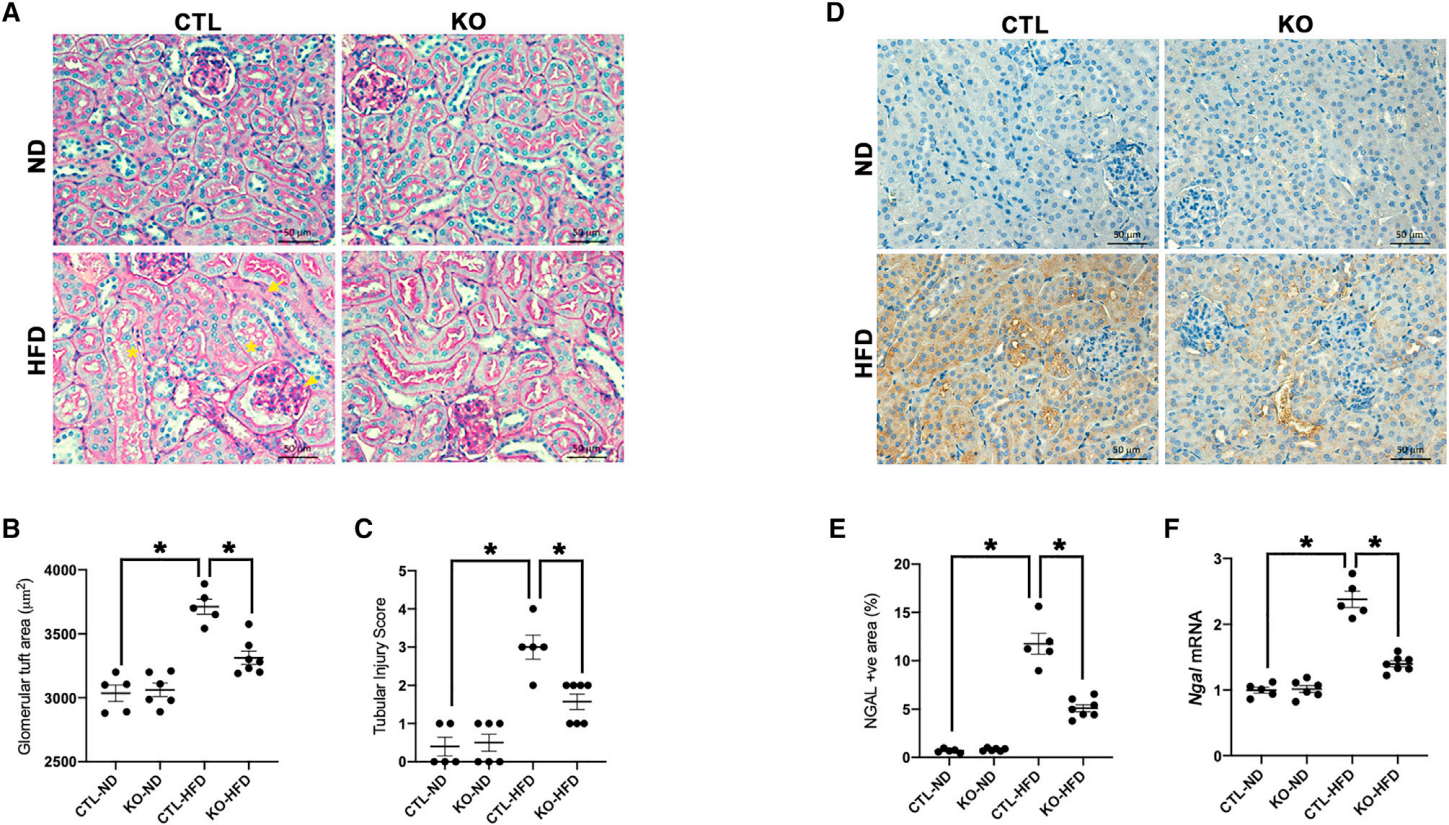
Tubule-specific *LincRNA-p21* deletion improves kidney histology in HFD-fed mice (A) PAS staining shows histological changes in *LincRNA-p21* KO mice or their CTL littermates fed with ND (KO-ND or CTL-ND) or HFD (KO-HFD, or CTL-HFD) (arrowhead, mesangial expansion; arrow, loss of brush border; star, cytoplasmic vacuolation). (B and C) Quantitative analysis of glomerular tuft area (B) and tubular injury score (C) by PAS staining at age 16 weeks. (D) IHC staining of NGAL in kidney after *LincRNA-p21* deletion. (E) Quantitative analysis of NGAL expression by IHC staining in kidneys. (F) Renal cortical expression of NGAL transcripts by qRT-PCR. Data are expressed as mean ± SEM from each group (n ≥ 5); ∗p < 0.05.

HFD-induced increase in serum creatinine (sCr), blood urea nitrogen (BUN), and urinary albumin levels in HFD-fed CTL mice were improved after tubular *LincRNA-p21* deletion (Figures 2F–2H). Periodic acid Schiff (PAS) staining (Figure 3A) demonstrates reduced kidney injury elicited by HFD in *LincRNA-p21* KO mice, as shown by ameliorated mesangial expansion (assessed by glomerular tuft area) and tubular injury score (Figures 3B and 3C). HFD increased gene and protein expression of the tubular injury marker neutrophil gelatinase-associated lipocalin (NGAL), which was ameliorated by tubular *LincRNA-p21* deletion (Figures 3D–3F). These findings suggest that *LincRNA-p21* deficiency enhanced the kidney's resilience to HFD-elicited injury.

### Tubule-specific *LincRNA-p21* deletion counteracted oxidative stress, inflammation, apoptosis, and ER stress

**Figure 4:**
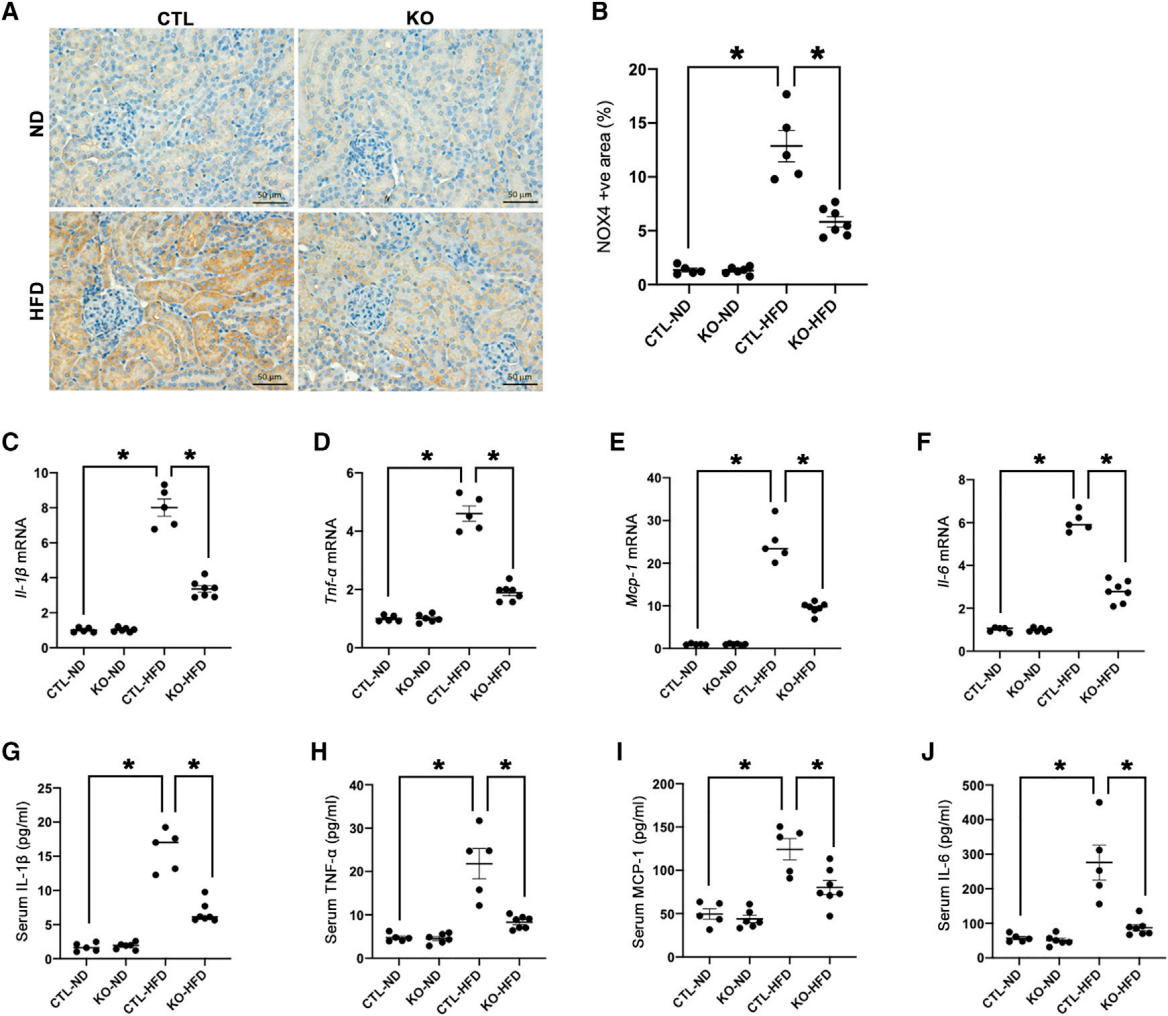
Tubule-specific *LincRNA-p21* deletion reduces oxidative stress and inflammation in HFD-fed mice (A) IHC staining of NOX4 in *LincRNA-p21* KO mice or their CTL littermates fed with ND (KO-ND or CTL-ND) or HFD (KO-HFD, or CTL-HFD). (B) Quantitative analysis of percentage NOX4-positive area by IHC staining in kidneys. qRT-PCR analysis showed that tubule-specific *LincRNA-p21* deletion reduced pro-inflammatory mediators *Il-1β* (C), *Tnf-α* (D), *Mcp-1* (E), and *Il-6* (F) in kidneys from HFD-fed mice. Serum pro-inflammatory mediators IL-1β (G), TNF-α (H), MCP-1 (I), and IL-6 (J) were reduced by tubule-specific *LincRNA-p21* deletion in HFD-fed mice. Data are expressed as mean ± SEM from each group (n ≥ 5); ∗p < 0.05.

**Figure 5:**
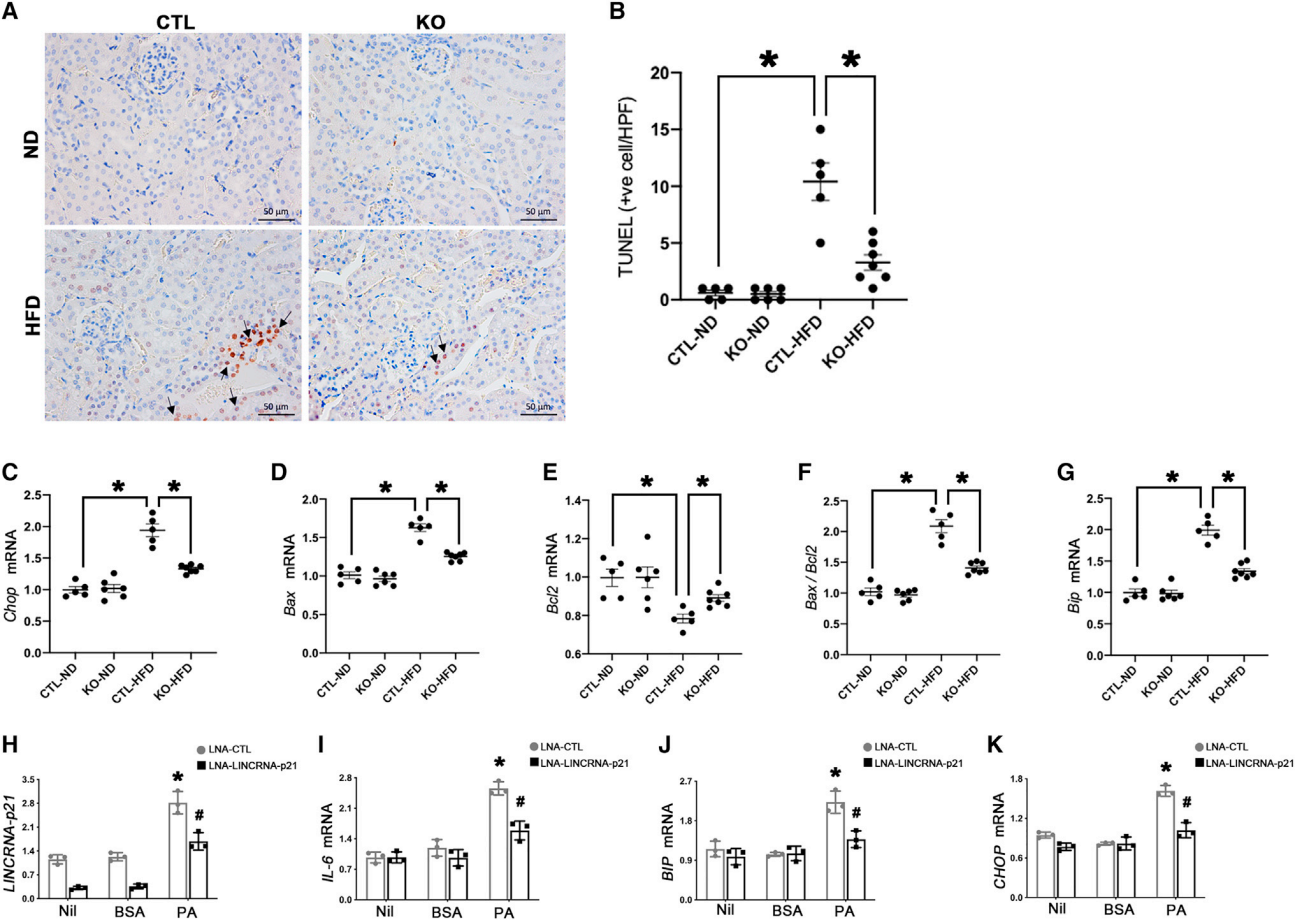
Tubule-specific *LincRNA-p21* deletion reduces apoptosis and ER stress in HFD-fed mice Representative micrographs of TUNEL assay (A) and quantification of apoptotic cells (arrows) in kidney (B). mRNA levels of apoptotic markers *Chop* (C), *Bax* (D), *Bcl2* (E), *Bax/Bcl2* ratio (F), and ER stress marker *Bip* (G) in kidneys from HFD-fed mice. Data are expressed as mean ± SEM from each group (n ≥ 5), ∗p < 0.05. Transcripts of *LincRNA-p21* (H)*, IL-6* (I), *BIP* (J), and *CHOP* (K) in HK-2 cells pretreated with *LNA-lincRNA-p21* GapmeR and stimulated by PA (500 μM) or BSA (0.475%) for 24 h. Results are expressed as mean ± SEM. Experiments were performed in triplicate. ∗p < 0.05 versus Nil, ^#^p < 0.05 versus LNA-CTL.

Nicotinamide adenine dinucleotide phosphate oxidase 4 (NOX4) is highly expressed in tubular epithelial cells of HFD-fed mice and was ameliorated by tubular *LincRNA-p21* deletion (Figures 4A and 4B). Tubule-specific *LincRNA-p21* deficiency significantly reduced the renal cortical expression (Figures 4C–4F) and systemic release (Figures 4G–4J) of pro-inflammatory cytokines including interleukin (IL)-1β, tumor necrosis factor alpha (TNF-α), monocyte chemoattractant protein (MCP-1), and IL-6. Terminal deoxynucleotidyl transferase-mediated dUTP nick end labeling of fragmented DNA (TUNEL) assay showed an increased number of apoptotic cells in CTL-HFD mice, while *LincRNA-p21* deletion decreased the apoptotic cell number in KO-HFD mice (Figures 5A and 5B). Furthermore, the transcript of pro-apoptotic marker C/EBP homologous protein (*Chop*), a marker of ER-stress-elicited apoptosis, was upregulated in CTL-HFD mice and reduced by *LincRNA-p21* deletion (Figure 5C). Likewise, upregulation of pro-apoptotic mediator B-cell lymphoma 2-associated X protein (*Bax*) and downregulation of anti-apoptosis mediator B-cell lymphoma 2 (*Bcl2*) were observed in CTL-HFD mice, and these alterations were partially reversed by *LincRNA-p21* deletion (Figures 5D–5F). In the renal cortex, mRNA level of ER stress marker binding immunoglobulin protein (*Bip*) was enhanced in CTL-HFD mice and reduced after *LincRNA-p21* deletion (Figure 5G). *In vitro*, *LINCRNA-p21* was increased by palmitic acid (PA) stimulation in HK-2 cells but partially reduced by silencing *LINCRNA-p21* (Figure 5H). Transcripts of the pro-inflammatory marker *IL-6*, as well as the ER markers *BIP* and *CHOP*, were elevated in HK-2 cells treated with PA but these changes were partially reversed by silencing *LINCRNA-p21* (Figures 5I–5K).

### HFD suppressed PI3K/AKT/mTOR/MDM2 signaling cascade to activate p53/*LincRNA-p21* axis in obese kidney

**Figure 6:**
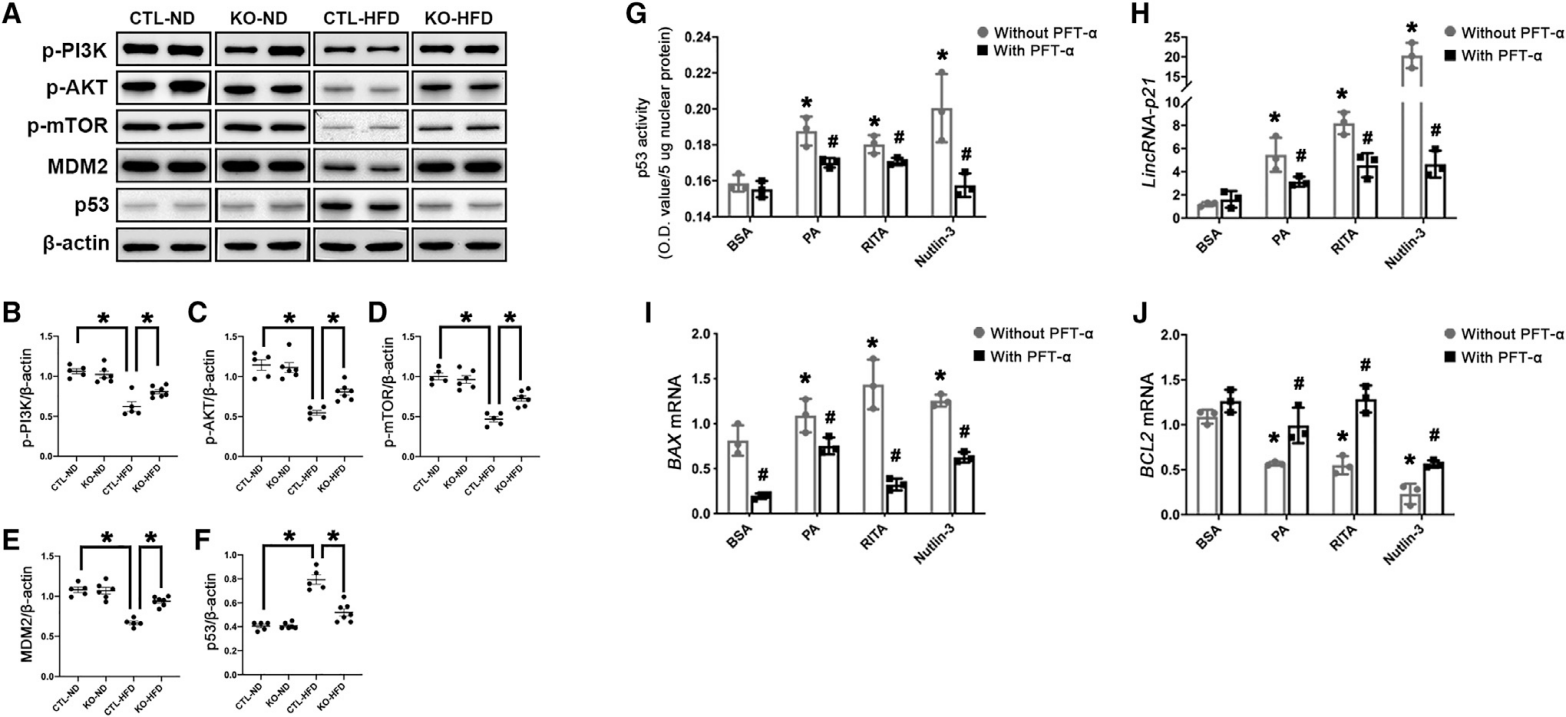
Activation of p53/LincRNA-p21 axis is regulated through PI3K/AKT/m-TOR/MDM2 signaling cascade *in vitro* and *in vivo* (A) Representative western blots showing phosphorylation levels of PI3K, AKT, mTOR, MDM2, and p53 in kidney tissues. Quantification of the expression of p-PI3K (B), p-AKT (C), p-mTOR (D), MDM2 (E), and p53 (F) by western blots, normalized to *β-ACTIN*. Data are expressed as mean ± SEM from each group (n ≥ 5), ∗p < 0.01. HK-2 cells pretreated with the p53 inhibitor PFT-α (5 μM) or vehicle were exposed to BSA (0.475%), PA (500 μM), or the p53 activators (RITA at 10 μM and Nutlin-3 at 20 μM) for 24 h, and p53 activity was measured by ELISA (G), and transcripts of *LincRNA-p21* (H), *BAX* (I), and *BCL2* (J) were examined by qRT-PCR. Results are expressed as mean ± SEM. Experiments were performed in triplicate. ∗p < 0.05 versus Nil, ^#^p < 0.05 versus LNA-CTL.

**Figure 7:**
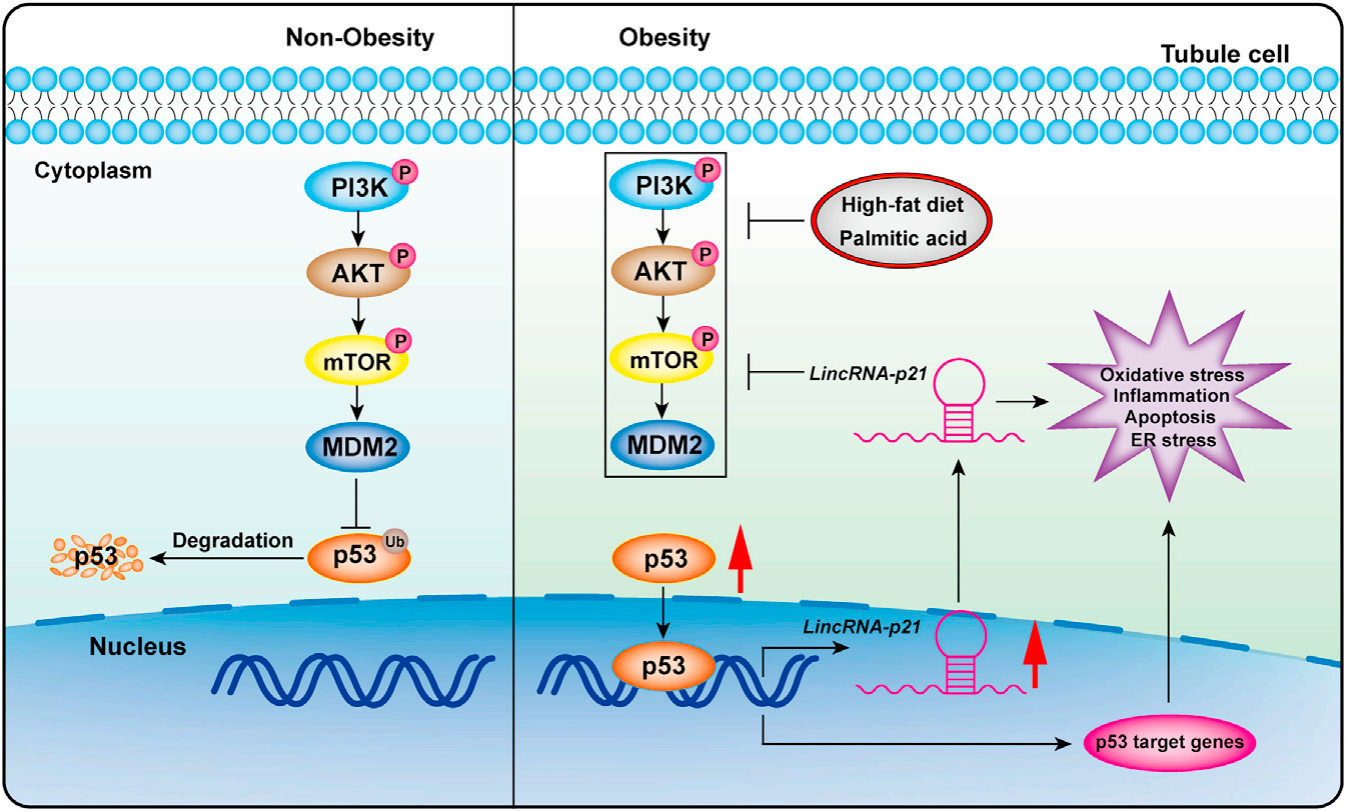
Schema of events and pathways of kidney injury mediated by lipotoxicity HFD or PA-elicited lipotoxicity inhibits PI3K/AKT/mTOR/MDM2 signaling to activate the downstream effectors p53/lincRNA-p21 that affect multiple pathological events, including oxidative stress, inflammation, apoptosis, and ER stress, in tubule cells during the development of obesity-related kidney lesions.

Western blot analysis demonstrated HFD suppressed the activation of the phosphatidylinositol 3-kinase (PI3K)/protein kinase B (AKT)/mechanistic target of rapamycin (mTOR) signaling cascade, a well-known upstream signaling pathway of p53, which resulted in reduced protein expression of murine double minute 2 homolog (MDM2), an endogenous inhibitor of p53, and eventually enhanced expression of p53 in obese kidney (Figures 6A–6F). Intriguingly, tubule-specific *LincRNA-p21* deletion reversed the suppression of the PI3K/AKT/mTOR signaling cascade by HFD, reinstating MDM2 and reducing p53 expression (Figures 6A–6F). *In vitro*, PA and the p53 activators (RITA and Nutlin-3) induced elevation in both p53 activity (Figure 6G) and *LINCRNA-p21* transcripts (Figure 6H) in HK-2 cells, while pretreating with the p53 inhibitor Pifithrin-α (PFT-α) partially reversed these elevations. Moreover, the elevated pro-apoptosis mediator *BAX* and suppressed anti-apoptosis mediator *BCL2* in HK-2 cells exposed to PA and p53 activators (RITA and Nutlin-3) were partially reversed by PFT-α (Figures 6I and 6J). Taken together, these findings proposed that HFD suppressed PI3K/AKT/mTOR/MDM2 signaling to motivate the p53/*LincRNA-p21* axis in obese kidney, while upregulated *LincRNA-p21* possibly further inhibited PI3K/AKT/mTOR/MDM2 signaling cascade and aggravated lipotoxic kidney injury to generate a vicious cycle (Figure 7).

## Discussion

The present study sheds light on the role of *LincRNA-p21*, a key mediator of multiple biological processes, in lipotoxicity-induced kidney damage. We show for the first time that lipotoxicity-induced kidney lesions were suppressed by deletion of *LincRNA-p21* in both cell culture and mouse models of DIO. Our findings provide convincing evidence that *LincRNA-p21* deficiency in kidney tubule cells protects the kidney from multiple HFD-induced pathological responses, including oxidative stress, inflammation and apoptosis, and ER stress. Mechanistically, our data demonstrate that lipotoxicity suppressed the PI3K/AKT/mTOR/MDM2 signaling cascade to enhance p53 transcriptional activity, which subsequently contributed to upregulation of *LincRNA-p21* in kidney tubule cells. Taken together, these results highlight a connection between *LincRNA-p21* and lipotoxicity-induced metabolic perturbation in tubule cells, corroborating a permissive role of *LincRNA-p21* in metabolic disease.

Accumulating evidence proposes lncRNA to be a possible cellular hub for the coordination of cellular processes involved in multifarious kidney disease progression, and strategies targeting multiple individual lncRNAs have received attention as potential treatments for kidney diseases such as diabetic kidney disease (DKD). Podocyte-specific lncRNA taurine-upregulated 1 overexpressing DKD mouse models improved glomerular phenotype in terms of albuminuria and histological changes through protecting podocytes from apoptosis and ER stress and alleviating extracellular matrix (ECM) accumulation.^11^, ^12^, ^13^, ^14^ More recent work suggests that two lncRNAs, Erbb4-IR^15^ and LRNA9884,^16^ promote DKD by enhancing kidney fibrosis and inflammation, respectively. Other lncRNAs, like metastasis-associated lung adenocarcinoma transcript 1,^17^, ^18^, ^19^ nuclear enriched abundant transcript 1,^20^^,^^21^ and zinc finger E-box-binding homeobox 1 antisense 1,^22^ have been implicated in DKD or the maintenance of lipid metabolism homeostasis by regulating multiple pathophysiological processes like inflammation, fibrosis, and ER stress. Understanding of lncRNA in obesity-related kidney injury is still very limited.

Emerging evidence unveils an integrative role of *LincRNA-p21* in numerous cellular functions, including modulation of cellular proliferation,^23^ apoptosis,^24^ cell-cycle arrest, ER stress,^25^ gene expression, protein stability, and inflammation,^26^ thus eliciting extensive biological actions in multiple pathological conditions, like inhibiting liver fibrosis,^27^^,^^28^ facilitating angiogenesis in lung cancer,^29^ preventing atherosclerosis by affecting smooth muscle cell proliferation and apoptosis,^30^ promoting liver fibrosis,^31^^,^^32^ facilitating apoptosis of hepatocellular carcinoma cells via activating ER stress,^25^ and inhibiting invasion and metastasis of hepatocellular carcinoma.^33^ These evidence proposes *LincRNA-p21* as a promising diagnostic and prognostic biomarker, as well as a therapeutic target, in human diseases. More importantly, it was found that *LincRNA-p21* silencing alleviated pathological changes in diabetic kidney, and reduced ECM in mouse mesangial cells stimulated by high glucose,^10^ indicating the involvement of *LincRNA-p21* in kidney disease. Mechanistically, *LincRNA-p21* acted as a molecular sponge for miR-18b that had inhibitory effects on connective tissue growth factor (CTGF) expression via interaction with the 3′-UTR of CTGF, ultimately enhancing expression of collagen I, collagen II, and fibronectin in mesangial cells.^10^ Such observations prompted us to investigate the role of *LincRNA-p21* in obesity-related kidney injury. Our findings for the first time highlighted a prominent role of *LincRNA-p21* in obese kidneys and PA-stimulated tubular epithelial cell pathological processes.

Tubular damage is an early predictor of future functional decline in type 2 diabetes, and initial stages of kidney damage in obesity or pre-diabetes are characterized by the presence of serum lipid abnormalities and ectopic renal lipid accumulation.^34^^,^^35^ Therefore, the identification of early metabolic changes of lipotoxicity in tubule cells could be critical in understanding the pathogenesis of obesity-related kidney injury or DKD. To explore the functional role of *LincRNA-p21*, we generated a tubule-specific *LincRNA-p21* KO mouse to unravel the potential of *LincRNA-p21* to become an early tubular damage marker as well as a therapeutic target in lipotoxic kidney injury. *LincRNA-p21* plays no regulatory role in lipid metabolism as its deletion did not affect serum lipid profile or BW in HFD-fed mice.

To elucidate the biological mechanisms through which *LincRNA-p21* mediates obesity-related kidney lesion, we assessed cell oxidative stress, inflammation, apoptosis and ER stress in kidney tissue from HFD-induced obese mice and cultured tubular cells under PA treatment. Lipotoxicity-elicited oxidative stress, inflammation, apoptosis, and ER stress were blunted in the absence of *LincRNA-p21*, and alleviated the associated kidney dysfunction. Intriguingly, we found that tubule-specific *LincRNA-p21* not only attenuated tubule injury but also contributed to reduced glomerular tuft area in HFD-induced obese mice. This finding reinforced the potential role of tubular injury in initiating loss of renal function with secondary sequelae that lead to glomerulosclerosis. The mostly accepted hypothesis of this phenomenon is that the overwhelming tubular glucose load due to increased plasma glucose levels during diabetes or pre-diabetes results in elevated proximal tubular sodium reabsorption through tubuloglomerular feedback, thus leading to a reduced afferent arteriolar vasoconstriction and subsequently glomerular hyperfiltration.^36^ Emerging evidence indicates that tubules may also contribute to glomerulopathy by secreting paracrine factors like nicotinamide mononucleotide and vascular endothelial growth factor (VEGF) that could diffuse back to the glomerulus to induce podocyte effacement, mesangial proliferation, and glomerular endothelial dysfunction in DKD.^37^^,^^38^

Others also reported an integrative functional role of *LincRNA-p21* in mediating various pathological responses.^10^^,^^39^ In particular, absence of *LincRNA-p21* resulted in a similar protective impact on a streptozotocin-induced DKD mouse model by reducing ECM in mesangial cells, alleviating kidney lesions, and hence improving renal function.^10^ In other words, *LincRNA-p21* regulates both lipotoxic and glycemic kidney injury, albeit through different functional mechanisms in different resident kidney cells. Therefore, *LincRNA-p21* may target multiple genes to regulate renal pathophysiology.

Our current data indicate that p53 is the upstream transcription factor that leads to *LincRNA-p21* induction in obesity-related kidney lesions, proposing a novel mechanism by which p53 participates in the progression of metabolic disease.^5^ Importantly, pharmacological inhibition of p53 suppressed *LincRNA-p21* expression and preserved its anti-apoptotic capability in PA-exposed renal tubule cells, consistent with previous findings.^40^^,^^41^ Here, we further showed that lipotoxicity directly suppressed the PI3K/AKT/mTOR/MDM2 signaling cascade to trigger p53 for its transcription of *LincRNA-p21* through *in vitro* and *in vivo* studies, while upregulated *LincRNA-p21* possibly further inhibits PI3K/AKT/mTOR/MDM2 signaling to enhance p53 activity to result in a positive feedback loop of injury. Although we did not highlight the likely downstream molecular mechanism mediated by *LincRNA-p21* during lipotoxic kidney injury, prior studies showed that the binding of *LincRNA-p21* with MDM2 enhanced p53 transcriptional activity in vascular smooth muscle cells from atherosclerotic mice,^30^^,^^39^ and that the overexpression of *LincRNA-p21* inhibited Wnt/β-catenin signaling in hepatic stellate cells,^42^ all supporting a crosstalk among *LincRNA-p21*, PI3K/AKT/mTOR, and MDM2/p53 signaling cascades. Recent data recognized *LincRNA-p21* as a molecular sponge to sequester microRNA-18b or microRNA-17-5p and promote the proliferation of mesangial cells exposed to high glucose or prevent activation of hepatic stellate cells,^10^^,^^42^ suggesting the classic mechanism that lincRNA as competitive endogenous RNAs or sponges of microRNAs to regulate gene expression could also be utilized by *LincRNA-p21* to reduce lipotoxic kidney injury. We and others observed the potential of *LincRNA-p21* to mediate p53 activity in a feedback manner,^30^^,^^39^ as well as the capacity of p53 in perturbing multiple biological processes under metabolic stress; however, we cannot exclude the possibility of *LincRNA-p21* as an effective upstream regulator of p53 to regulate its function under lipotoxic kidney injury. This hypothesis merits future research for a better understanding of the metabolic functions and molecular mechanisms of p53 family members.

Collectively, lipotoxicity suppressed the PI3K/AKT/mTOR/MDM2 signaling pathway to activate the downstream effectors p53/*LincRNA-p21*, which trigger subsequent kidney injury. Tubule-specific ablation of *LincRNA-p21* in kidney protected HFD-induced kidney injury structurally and functionally through ameliorating oxidative stress, inflammation, apoptosis, and ER stress. Our findings unraveled the potential of the tubular p53/*LincRNA-p21* signaling axis in the regulation lipotoxicity-induced pathological cellular events, and opens a new avenue for developing treatment of lipotoxicity-elicited kidney injury.

## Materials and methods

### Chemicals and reagents

Dulbecco's modified Eagle’s medium/Nutrient Mixture F-12 (DMEM/F12), GlutaMAX, 100 IU/mL penicillin, 100 μg/mL streptomycin, fetal bovine serum (FBS), and RNAiMAX reagent were purchased from Invitrogen (Carlsbad, CA). Custom-made locked nucleic acid (LNA) GapmeR antisense oligonucleotides targeting *LincRNA-p21* (LNA-LincRNA-p21) and corresponding negative CTL (LNA-CTL) were purchased from Qiagen (Germantown, MD), and Opti-MEM Reduced serum medium was obtained from Applied Biosystems (Carlsbad, CA). MDM2 inhibitor Nutlin-3, p53 inhibitor PFT-α, and p53 activator RITA (NSC 652287) were obtained from Selleckchem (Houston, TX). Fatty-acid-free BSA and PA were obtained from Sigma-Aldrich (St Louis, MO). PA was conjugated with BSA before being added into culture medium. In brief, 10% fatty-acid-free BSA solution dissolved in phosphate-buffered saline (PBS) was heated to 37°C before the addition of a 200 mM PA stock solution dissolved in absolute ethanol to gain a 10 mM working concentration, while a comparable concentration of 0.475% BSA was used as vehicle CTL for PA. All the other chemicals and reagents utilized in this study, unless otherwise specified, were obtained from Sigma-Aldrich.

### Generation of tubule-specific *LincRNA-p21* KO mice

All animal procedures were performed following the National Institutes of Health Guidelines for the Humane Treatment of Animals, with approval from the Committee on the Use of Live Animal in Teaching and Research of the University of Hong Kong. Diabetic *db/db* mice and the lean CTL *db/m* mice were obtained from The Jackson Laboratory (Main Street Bar Harbor, ME). The *Ksp-CreER*^*T2*^ mice were obtained from Professor Peter Igarashi (The UT Southwestern O'Brien Kidney Research Core Center, Dallas, TX) and the heterozygous *LincRNA-p21*^*fl/wt*^ mice were bought from the Jackson Laboratory (Main Street Bar Harbor, ME). For the *Ksp-CreER*^*T2*^ mice, CreER^T2^ fusion protein expression is regulated exclusively under the kidney specific *Ksp-Cadherin* (*Cadherin 16*) gene promoter, thus the *Ksp-CreER*^*T2*^ fusion protein would specifically be found in the renal tubular epithelial cells upon activation by 4-hydroxytamoxifen. The *LincRNA-p21*^*fl/fl*^ mouse carries two *loxP* sites sandwiching the *LincRNA-p21* sequence. By cross-breeding *Ksp-CreER*^*T2*^ and *LincRNA-p21*^*fl/fl*^ mice, we successfully generated the tubule-specific *LincRNA-p21* KO, which carried both the *Ksp-CreER*^*T2*^ and *LincRNA-p21*^*fl/fl*^ alleles (Figure S1). These mice without *Ksp-CreER*^*T2*^ allele served as the CTL. At the age of week 6, 4-hydroxytamoxifen (1 mg/10 g BW) was administrated to all experimental mice (four groups, five to seven mice in each group) by daily intraperitoneal injection for five consecutive days to induce the *Cre/loxP* recombination in kidney tubules. qRT-PCR and FISH using Stellaris RNA FISH probe and reagents (LGC Biosearch Technologies, CA) were used to confirm the successful deletion of *LincRNA-p21* (Figures 1D and 1E).

To establish DIO in KO and CTL mice, mice at week 8 were fed with HFD (60 kcal% fat, D12492, Research Diet Inc., NJ) for 8 weeks, whereas mice fed with ND (10 kcal% fat, D124350B, Research Diet Inc., NJ) served as CTL. BW was monitored biweekly. Blood was collected from tail vein following overnight fasting for renal function and biochemical analyses. Serum glucose levels were determined by Accu-Check Aviva glucometer (Roche Diabetes Care, Mannheim, Germany). Serum sCr, urinary creatinine, BUN, serum triglycerides, and cholesterol levels of mice were measured by enzymatic method (Stanbio Laboratory; St Boerne, TX). Urinary albumin was determined using an ELISA quantification kit (Bethyl Laboratories, TX). All mice were sacrificed at the end of experiments and kidneys were removed for histological analyses. Markers of oxidative stress, inflammation, apoptosis, and ER stress were evaluated by qRT-PCR, western blot analysis, and immunohistochemical (IHC) staining.

### RNA isolation and qRT-PCR

Total RNA was isolated from HK-2 cells using NucleoSpin RNA II total RNA Isolation Kit (Macherey-Nagel, Duren, Germany). NucleoSpin TriPrep Kit (Macherey-Nagel) was used to extract total RNA from mouse renal cortex. Isolated RNA (2 μg) was utilized for synthesizing cDNA by High-Capacity cDNA Reverse Transcription Kit (Applied Biosystems). qRT-PCR analyses were conducted in the StepOnePlus Real-time PCR Systems (Applied Biosystems) using SYBR Green Master Mix (Applied Biosystems) and specific primers (Table S1). Relative quantification of genes in each individual sample was normalized to β-actin expression and all experimental groups were compared with their respective CTL groups using StepOne software v2.3 (Applied Biosystems).

### Western blot analysis

HK-2 cells were collected and washed with cold PBS after experiments and solubilized with lysis buffer (1% Triton X-100, 0.1% SDS, 0.5% sodium deoxycholate, 50 mM Tris-HCl, 150 mM NaCl, 5 mM EDTA) containing protease inhibitor cocktail (Thermo Scientific, CA). Total protein from mouse kidney cortex was isolated by NucleoSpin TriPrep Kit (Macherey-Nagel). Protein concentrations from the cultured cells or cortical lysates were detected by the BCA Protein Assay Reagent (Thermo Scientific). Equal amounts of proteins were separated on 4%–12% precast Bis-Tris gel (Bio-Rad, Hercules, CA) and then transferred from the gel to a 0.45-μm polyvinylidene difluoride membrane (Millipore, Bedford, MA) using standard electroblotting procedures. After blocking with 5% non-fat milk in Tris-buffered saline with Tween 20 (10 mM Tris-HCl, pH 7.6, 150 mM NaCl, 0.05% Tween 20), the blots were incubated with primary antibody overnight, washed with Tris-buffered saline with Tween 20, and then incubated with horseradish peroxidase-conjugated secondary antibody for 2 h and subsequently processed for incubation with enhanced chemiluminescence (ECL) prime chemiluminescence (GE healthcare, Buckinghamshire, UK) and visualized by ChemiDoc XRS+ system (Bio-Rad). Intensity of the protein bands was measured via Image Lab software (Bio-Rad).

### Morphometric analysis of the mice kidney sections

Kidney samples were fixed in 10% formalin, embedded in paraffin, and sectioned to 4-μm thickness. Morphologic analyses of the kidney sections were carried out after PAS staining (Sigma). Injured tubules in the renal cortex were assessed by the presence of necrosis, loss of brush borders, vacuolation, cast formation, and tubular dilatation. Tubular injury was scored from 0 to 5 according to the percentage of injured tubules (0, normal; 1, tubular lesion <10%; 2, 10%–20% lesion; 3, 20%–30% lesion; 4, 30%–50% lesion; 5, >50% lesion) from 10 randomly chosen, nonoverlapping fields at 400× magnification under a microscope. Glomerular tuft area was quantified by ImageJ software (National Institutes of Health, United States) from 20 randomly selected cortical glomeruli at 400× magnification and data were expressed as average μm2 per glomerulus. Only glomeruli containing a visible vascular or urinary pole were considered for area measurements.

### Immunohistochemistry studies and apoptosis determination

*In situ* detection of apoptosis was performed using the ApopTag Peroxidase Kit (EMD Millipore, St Louis, MA) based on TUNEL staining, and data were expressed as number of apoptotic cells per high-power field. For IHC staining, paraffin-embedded kidney sections were deparaffinized and rehydrated, followed by microwave-based antigen retrieval in citrate buffer (10 mM), pH 6.0, or protease K solution (20 μg/mL) in Tris-EDTA buffer, pH 8.0. Sections were quenched by 3% hydrogen peroxide and blocked with 2% BSA. Primary antibodies against NGAL and NOX4 (Abcam, Cambridge, MA) were applied on the sections overnight, followed by incubation with peroxidase-conjugated second antibodies (Dako, Glostrup, Denmark). Sections were developed using 3,3′diaminobenzidine substrate (Dako) and counterstained with hematoxylin. Ten images per section were captured in a blinded manner. Quantification of staining was performed using ImageJ analysis software. Data were expressed as percentage of positively stained area.

### Measurement of serum IL-β, TNF-α, IL-6, and MCP-1

Pro-inflammatory cytokines including IL-1β, IL-6, TNF-α, and MCP-1 in serum from all mice were measured in a single batch by Milliplex multiplex assays (EMD Millipore) according to the manufacturer's instructions and using a Luminex 200 multiplex detection system (EMD Millipore).

### Cell culture

The human proximal tubular cell line, human kidney 2 (HK-2) cells, was purchased from American Type Culture Collection (ATCC, Manassas, VA) and cultured in DMEM/F12 fermented with 10% FBS, 1% penicillin/streptomycin at 37°C in 5% CO_2_. Cells were growth arrested in culture medium containing 0.5% FBS for 12 h before the initiation of all experiments.

### LNA antisense oligonucleotides transfection in HK-2 cells

*In vitro* knockdown experiments were performed by using specific LNA GapmeR antisense oligonucleotides (ASOs). Three custom LNA GapmeR ASOs targeting *LINCRNA-p21* (LNA-*LINCRNA-p21*: 5′-CTTGGCTGGCGGAAGG-3′, 5′-CTTGGCTGGCGGAAGG-3′ 5′-CTTGGCTGGCGGAAGG-3′) and one LNA GapmeR ASO targeting negative CTL (LNA-CTL: 5′-AACACGTCTATACGC-3′) were used. One day before transfection, cells were plated in medium without antibiotics and FBS in a 12-well plate at a density that resulted in 60%–70% cell confluence. LNA-*LINCRNA-p21* and LNA-CTL were diluted to the required concentration in Opti-MEM and mixed with Lipofectamine RNAiMAX, followed by incubation for 10 min at room temperature and then added to each well. Knockdown efficiency was determined 24 h after transfection by qRT-PCR, and cells with knockdown efficiency >70% were used for subsequent experiments.

### Measurement of p53 activity

p53 activity in HK-2 cells with different treatments was measured using a p53 Transcription Factor Activity Assay Kit (BioVision, Mountain View, CA) according to the manufacturer's specifications. This kit is a non-radioactive assay for the quantitative measurement of specific transcription factor DNA binding activity of active p53, and a biotin-labeled p53 response element DNA binds specifically to active p53 in nuclear extract and forms a complex that could be detected by the p53 specific monoclonal antibody. In brief, 5 μg of nuclear extract samples from HK-2 cells subjected to different culture conditions and CTLs were pipetted into wells precoated with a biotin-labeled p53 response element DNA that captures specifically active p53 to form a complex, which was then detected by adding anti-p53 monoclonal antibody that is conjugated to horseradish peroxidase streptavidin after removing unbound material by washing with washing buffer. Horseradish peroxidase catalyzed the conversion of a chromogenic substrate (tetramethylbenzidine) to a colored solution with color intensity proportional to the amount of protein present in the sample. Spectrophotometric data were expressed by measuring the absorbance at 450 nm using a microplate reader. Quantitative results were calculated from at least three independent biological experiments.

### Statistical analysis

Statistical analysis was performed using Prism software version 7.0 (GraphPad, San Diego, CA). All data were expressed as mean ± SEM. Two-tailed Student's t test or one-way ANOVA followed by a post hoc multiple comparison (Tukey) test was carried out where applicable. p < 0.05 was considered statistically significant.

## Supplemental information

Document S1. Figure S1 and Table S1

Document S2. Article plus supplemental information
