# Supplementary material for: Tubule-specific deletion of LincRNA-p21ameliorates lipotoxic kidney injury
Source: Mol Ther Nucleic Acids. 2021 Nov 4;26:1280–90. doi: 10.1016/j.omtn.2021.10.029 (PMC8609107; doi:10.1016/j.omtn.2021.10.029)
Supplement: Document S1. Figure S1 and Table S1 [file mmc1.pdf]

OMTN, Volume *26*

Supplemental information

Tubule-specific deletion of *LincRNA-p21* ameliorates lipotoxic kidney injury

Bin Li, Joseph C.K. Leung, Loretta Y.Y. Chan, Hong-Yu Li, Wai-Han Yiu, Sarah W.Y. Lok, Rui Xue, Yi-Xin Zou, Wei Chen, Kar-Neng Lai, and Sydney C.W. Tang

Figure S1

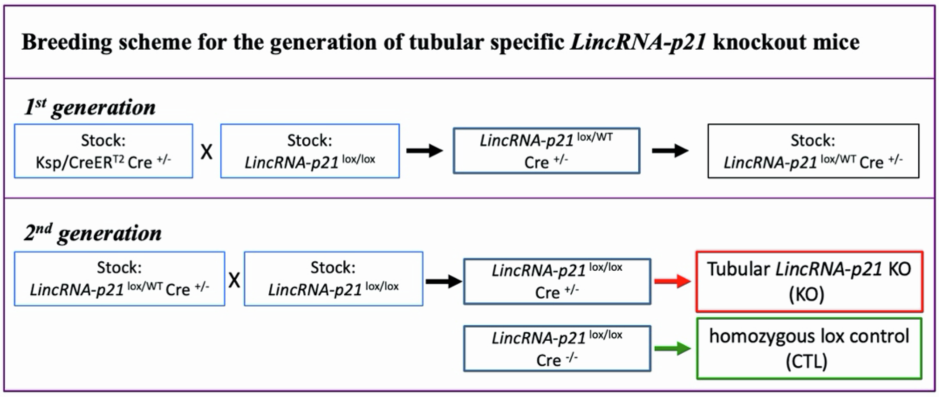

Figure S1. **Breeding strategy of and experimental protocol with *LincRNA-p21* KO mice.**

(A) Generation of tubule-specific *LincRNA-p21* knockout mice. (B) Timeline & protocol for the establishment of diet induced obesity (DIO) model.

Table S1. Custom primers for quantitative real-time PCR.

Gene	Species	Forward 5'-3'	Reverse 5'-3'
BAX	Human	TCTGACGGCAACTTCAACTG	CACTGTGACCTGCTCCAGAA
BCL2	Human	ATGTGTGTGGAGAGCGTCAA	ACAGTTCCACAAAGGCATCC
BIP	Human	TGTTCAACCAATTATCAGCAAACTC	TTCTGCTGTATCCTCTTCACCAGT
CHOP	Human	AGAACCAGGAAACGGAAACAGA	TCTCCTTCATGCGCTGCTTT
IL-6	Human	ATGAACTCCTTCACAAG	TGTCAATTCGTTCTGAAGAG
LINCRNA-p21	Human	GGGTGGCTCACTCTTCTGGC	TGGCCTTGCCCGGGCTTGTC
β-ACTIN	Human	CGGGAAATCGTGCGTGACAT	GAACTTTGGGGGATGCTCGC
Bax	Mouse	CCAAGAAGCTGAGCGAGTGTCT	AGCTCCATATTGCTGTCCAGTTC
Bcl2	Mouse	TTGTAATTCATCTGCCGCCG	AATGAATCGGGAGTTGGGGT
BiP	Mouse	TCATCGGACGCACTTGGAA	CAACCACCTTGAATGGCAAGA
Chop	Mouse	GCAGCGACAGAGCCAGAATAA	TTCTGCTTTCAGGTGTGGTGG
Il-1b	Mouse	CCTTCCAGGATGAGGACATGA	AACGTCACACACCAGCAGCTT
Il-6	Mouse	CATGTTCTCTGGGAAATCGTGG	AACGCACTAGGTTTGCCGAGTA
LincRNA-p21	Mouse	CCTGTCCACTCGCTTTC	GGAACTGGAGACGGAATGTC
Mcp-1	Mouse	TTGACCCGTAAATCTGAAGCTAAT	TCACAGTCCGAGTCACACTAGTTCAC
Ngal	Mouse	AATGTCACCTCCATCCTG	ATTTCCCAGAGTGAACTG
Tnf-a	Mouse	CGCTCTTCTGTCTACTGAACTT	GATGAGAGGGAGGCCATT
β-Actin	Mouse	CGCCACCAGTTCGCCATGGA	TACAGCCCGGGGAGCATCGT
